# Changes in Quality and Collagen Properties of Cattle Rumen Smooth Muscle Subjected to Repeated Freeze—Thaw Cycles

**DOI:** 10.3390/foods11213338

**Published:** 2022-10-24

**Authors:** Yinjuan Cao, Zhaoyang Song, Ling Han, Qunli Yu, Xiangying Kong, Shibao Li

**Affiliations:** 1 College of Food Science and Engineering, Gansu Agricultural University, Lanzhou 733070, China; 2 Haibei State Institute of Animal Husbandry and Veterinary Science, Haibei 812299, China; 3 Qinghai Cocosili Biological Engineering Co., Ltd., Xining 810100, China

**Keywords:** cattle rumen smooth muscle, freeze-thaw, collagen, structural and functional properties, quality

## Abstract

This study revealed changes in the quality, structural and functional collagen properties of cattle rumen smooth muscle (CSM) during F-T cycles. The results showed that thawing loss, pressing loss, β-galactosidase, β-glucuronidase activity, β-sheet content, emulsifying activity index (EAI), emulsion stability index (ESI), surface hydrophobicity, and turbidity of samples were significantly (*p* < 0.05) increased by 108.12%, 78.33%, 66.57%, 76.60%, 118.63%, 119.57%, 57.37%, 99.14%, and 82.35%, respectively, with increasing F-T cycles. Meanwhile, the shear force, pH, collagen content, α-helix content, thermal denaturation temperature (T_max_), and enthalpy value were significantly (*p* < 0.05) decreased by 30.88%, 3.19%, 33.23%, 35.92%, 10.34% and 46.51%, respectively. Scanning electron microscopy (SEM) and SDS-PAGE results indicated that F-T cycles induced an increase in disruption of CSM muscle microstructure and degradation of collagen. Thus, repeated F-T cycles promoted collagen degradation and structural disorder in CSM, while reducing the quality of CSM, but improving the functional collagen properties of CSM. These findings provide new data support for the development, processing, and quality control of CSM.

## 1. Introduction

With the rapid development of the livestock breeding industry, many livestock by-products have been produced during the slaughtering and meat processing of livestock [1]. At present, the low utilization of livestock by-products is causing economic losses. If the meat value of smooth muscle can be improved, the output value of the cattle industry can be increased and the healthy development of the cattle and food industries can be promoted [2]. Therefore, these livestock by-products should be appropriately developed and utilized effectively to increase the economic benefits of livestock slaughtering and processing enterprises, avoid environmental pollution and resource waste, and enhance the construction of a circular economy for livestock by-products, thereby promoting green, healthy, and sustainable development. 

The rumen is the main by-product of cattle, accounting for 80% of the entire gastric chamber, of which smooth muscle accounts for 40–50% [3]. Cattle rumen smooth muscle (CSM) is a kind of muscle tissue that is widely distributed in the stomach, intestines, and other internal organs of animals. CSM is rich in nutrients including proteins, fats, calcium, phosphorus, iron, and thiamin [2]. However, CSM differs from skeletal muscle in terms of protein composition and structure. Collagen, which is the main structural and functional component of smooth muscle, is 10% more abundant in the rumen wall than in skeletal muscle.

CSM is widely consumed in several Asian countries because of its favorable texture. Typically, CSM is frozen for storage, transport, and marketing [1]. Nevertheless, inadequate cold chain technology and high-temperature fluctuations can affect meat quality, including mechanical protein denaturation, damage to tissues, discoloration, increased lipid oxidation, and water loss after thawing [4]. Repeated freeze–thaw cycles have been found to cause recrystallization, leading to an increase in the volume of ice crystals, damaging the cell membrane structure and the tissue structure, and accelerating oxidative denaturation of protein, and water loss [5]. Boonsumrej et al. found that the solubility of shrimp protein decreased, the muscle fiber gap increased, and the shear force increased with the increase of freeze–thaw (F-T) cycles [6]. Zhou et al. reported that the hydroxyl free radicals produced by oxidation reactions in muscle can also induce the oxidation of muscle fibrin, which leads to denaturation and degradation [7]. Strange et al. found that with the increase in freezing and thawing times, the organoleptic quality of pork liver decreased, possibly due to the high collagen content in pork liver [8]. In general, the effect of repeated F-T cycles on meat quality varies with muscle and protein types. However, the quality stability of CSM during F-T cycles and the biochemical processes that lead to changes in quality remain unknown. 

In our study, the effects of repeated F-T cycles on changes in CSM quality, collagen function and structure are explored and the effects on CSM quality of changes in collagen properties are further discussed. This paper provides a reference for better development and utilization of livestock by-product resources and smooth muscle quality control.

## 2. Materials and Methods

### 2.1. Sampling

A total of nine healthy local yellow cattle (live and carcass traits are shown in Table 1) from Kang Mei Meat Co., Ltd. (Kangle, China) were slaughtered according to the standard procedure (GB/T 19477-2018). Cattle rumen is connected to the oesophagus at the anterior end and to the duodenum at the posterior end. The nine rumen were removed immediately after slaughter, washed with distilled water to remove internal contamination, and immediately transferred to the laboratory in an icebox. Cattle rumen smooth muscle (CSM) was obtained by removing the villi and mucous layer from the surface and removing the visible fat, then the muscles were cut into 10 × 10 cm pieces, and all CSM samples were packed in polyethylene bags [2]. Six CSM samples were randomly selected as the controls (without freeze–thaw cycles). The remaining samples were frozen in a −25 °C refrigerator until the core temperature reached −18 °C, before frozen storage. The samples frozen were thawed in a 4 °C refrigerator for 12 h until the core temperature reached 4 °C, at which point the first F-T process was accomplished. The second, third, fourth, and fifth F-T cycles were completed successively by the above method. F_0_, F_1_, F_2_, F_3_, F_4,_ and F_5_ represent samples subjected to up to five F-T cycles, respectively. All chemicals were from Aladdin (Shanghai, China), and analytical or higher grades were used.

### 2.2. Thawing Loss and Pressing Loss

The thawing loss of CSM was determined according to the method described by Xu et al. [9] and calculated as:

$$\mathrm{Thawing}\;\mathrm{loss}\left(\%\right)=\frac{m_{1}{\;-\;m}_{0}}{m_{1}}\times 100\%$$

where *m*_1_ is the sample weight before thawing and *m*_0_ is the sample weight after thawing.

Pressing loss was determined using a pressure loss rate meter (YBW-2, Shanghai Soil Instrument Co., Ltd., Shanghai, China) at a pressure of 341 N for 5 min [10] and expressed as:

$$\mathrm{Pressing}\;\mathrm{loss}\;\left(\%\right)=\frac{m_{3}{\;-\;m}_{2}}{m_{3}}\times 100\%$$

where *m*_3_ is the sample weight before pressing and *m*_2_ is the sample weight after pressing.

### 2.3. Shear Force

The samples were placed in PA plastic cooking bags and cooked in a water bath at 75 °C for 20 min, until the central temperature reached 75 °C. Each sample was cut into 50 × 10 mm^2^ pieces (with the fiber axis oriented at 50 mm) and then stacked 2–3 pieces to obtain 10 mm thick (standard thickness: 10 mm) samples. Subsequently, each sample was placed on a texture analyzer (Isenso, Brookfield Corporation, Atlanta, GA, USA) and tested on a Warner-Bratzler v-shear apparatus [2]. The crosshead speed was 1.5 mm/s, and the pressure measuring element was 50 kg. Three samples were analyzed for each treatment. 

### 2.4. pH

The pH of CSM was measured with a digital pH meter (PB-10, Shaanxi Tian Yun Instrument Manufacturing Co., Ltd., Shanxi, China), as described by Xu et al. [9].

### 2.5. Microstructure Analysis

The microstructure of CSM was determined using the method described by Cao et al. [2]. Samples were fixed in 2.5% glutaraldehyde at 4 °C for 72 h and then washed with 0.1 M phosphate buffer. They were fixed with 1% osmolarity solution for 2 h. After dehydration with graded ethanol, samples were plated with gold and observed by scanning electron microscope (Quanta 450 FEG, FEI Co., Hillsborough, OR, USA) with an accelerating voltage of 20 kV.

### 2.6. Collagen Extraction

The rumen samples were cut into pieces and put into a 500 mL Erlenmeyer flask, then soaked in the 20-fold volume of 0.1 mol/L NaOH solution for 20 h to remove pigment and miscellaneous protein. The samples were washed with ultrapure water to neutral pH, and then a 30-fold volume of 10% n-butanol was added. Each sample was soaked for 24 h to remove excess fat and rinsed with distilled water until reaching neutral pH. Then, 10 g of the treated sample was weighed and placed in a 500 mL glass flask with 11 times the volume of distilled water. The pH was adjusted to 2.0 with a lactic acid solution. Subsequently, 3% pepsin (1:3000) was added to extract the collagen, the mixture was shaken continuously with a shaker for 24 h, and then filtered. The filtrate was collected and subjected to salting out, centrifugation, dialysis, and vacuum freeze-drying, and then the obtained collagen was placed in a refrigerator at −18 °C before use.

### 2.7. Collagen Content and Solubility

The content of collagen in the CSM was determined using the method described by Mikolajczak et al. for the determination of hydroxyproline [11]. A 5 g sample of minced muscle tissue was homogenized with 8 mL of Ringer’s solution. Next, the samples were heated in a water bath at 77 °C for 60 min. The resulting solution was centrifuged for 20 min (5000 r/min) in an MPW360 centrifuge. The supernatant and the precipitate were hydrolyzed in 6 M HCl for 16 h, respectively. The hydrolysate was filtered and reacted with p-dimethyl benzaldehyde to determine the hydroxyproline content [12]. The result was converted into soluble collagen (supernatant) and insoluble collagen (sediment) by multiplying the result by 7.25, and the total collagen was the sum of the two fractions. The solubility of collagen was expressed as the percentage of soluble collagen to total collagen.

### 2.8. Collagen Degradation Analysis

The activities of β-galactosidase and β-glucuronidase were determined according to the instructions of the ELISA kit from Shanghai Yuanmu Biotechnology Co., Ltd (Shanghai, China). The standard sample was diluted to 150 U/L, 100 U/L, 50 U/L, 25 U/L, and 12.5 U/L with the standard diluent to prepare the standard curve. Briefly, 1 g sample was weighed, and then mixed with 9 mL PBS buffer (pH 7.4), homogenized with a homogenizer (YJQ, Xinrui, Shanghai, China), and centrifuged at 5000 r/min for 20 min. The blank and the sample were set on the enzyme plate. The reagents were added to each well according to the instructions and mixed uniformly. The absorbance values of the samples were measured after color development at 37 °C for 15 min.

According to the method described by Maqsood et al. [13], freeze-dried samples of cattle rumen collagen obtained from different F-T cycles were subjected to SDS-PAGE analysis. Then, 5% SDS was used to solubilize collagen and the samples (20 µg collagen) were loaded onto the polyacrylamide gel comprising 8% separating gel and 5% stacking gel. Collagen identification was based on the molecular weight and clarity of the retained protein bands. A wide range of molecular weight markers was used for the estimation of protein molecular weight, from 245 KDa to 5 KDa.

### 2.9. Collagen Secondary Structure Analysis

The secondary structure of the cattle rumen collagen was determined by Fourier transform infrared spectrometer (FTIR, IS10, Thermo Nicolet Corporation, Madison, WI, USA) according to the process stated by Li et al. [10]. Freeze-dried collagen (2 mg) samples were scanned using FTIR (4000–400 cm^−1^). The spectra in the selected amide I bands (1600–1700 cm^−1^) were analyzed by PeakFit 4.12 software, and second-order derivative peaks were fitted until the iteration was invariant. The relative percentages of the areas of the subpeaks were obtained from the peak summary data, and the percentage was calculated for each component within the secondary structure of the collagen. The Fourier spectrum of the collagen was plotted by Origin 9.0 software. Each sample was measured three times and the average values calculated.

### 2.10. Collagen Thermal Stability Analysis

DSC analysis of dried samples of bovine rumen collagen was carried out using a differential scanning calorimeter (DSC-1, Mettler Toledo, Switzerland) [14]. Approximately 4 mg of protein powder was weighed into an aluminum pan, and a sealed empty aluminum pan was used as the reference. The run was conducted in the temperature range of 20–180 °C in a nitrogen stream (heating rate: 5 °C/min, flow rate: 20 mL/min). 

### 2.11. Collagen Emulsion Property, Turbidity Analysis

The emulsifying activity index (EAI) and emulsion stability index (ESI) were determined according to method described by Chan et al. with some small amendments [15]. Soybean oil and 1% collagen solution (0.1 mol/L acetic acid solution) were put into a 50 mL centrifuge tube at 1:3 (*v*/*v*) and emulsified in a homogenizer (FJ200-SH, Shanghai Specimen and Model Factory, Shanghai, China) at 10,000 r/min for 1 min. The resulting solutions were emulsified at 0 and 10 min, respectively, and 200 μL of the emulsion was pipetted from the bottom of a centrifuge tube into a test tube, diluted to 10 mL with 0.1% SDS (pH 7.0), and the absorbance value of the emulsion at 500 nm was measured. The calculation is as follows:

$${\mathrm{EAI}(m}^{2}/g)=\frac{2\times 2.303}{C\times 0.001{\times (1-\varphi )\times 10}^{4}}{\times A}_{0}\times \mathrm{dilution}$$

$$\mathrm{ESI}\left(\%\right)=\frac{A_{10}}{A_{0}}\times 10$$

where *A*_0_ and *A*_10_ are absorbance of the emulsion at 0 min and 10 min, respectively; C is concentration of sample before emulsification (mg/mL); φ is volume fraction occupied by soybean oil (*v*/*v*).

Surface hydrophobicity of CSM collagen was quantified according to the method stated by Zhu et al. [16]; a collagen solution of 1.0 mg/mL was prepared with 0.5 mol/L acetic acid then mixed with 10 μL of 1-anilinonaphthalene-8-sulfonic acid (ANS). After 15 min of light-avoidance reaction, the fluorescence intensity was measured with a fluorescence spectrometer at 371 nm (excitation wavelength) and 467 nm (emission wavelength), at a scan rate of 600 nm/min. The slit width was 10 nm. The protein concentration was plotted against the fluorescence intensity and the slope represented the surface hydrophobicity index.

CSM collagen turbidity was measured by the method of Ran et al. [17]. The lyophilized collagen powder was prepared as a 5 mg/mL solution with 0.5 mol/L acetic acid, then placed in a water bath at 30 °C for 30 min. The resulting solution was cooled naturally to room temperature and the absorbance value was measured at 310 nm. The acetic acid solution was used as blank control. The turbidity was expressed as A_310_.

### 2.12. Statistical Analysis

Statistical analyses were carried out using IBM SPSS version 22.0 software (SPSS, Inc., Chicago, IL, USA). Graphs were constructed using Origin 8.6 software. The results were expressed as means of three independent experiments. All data were presented as mean ± standard deviation (SD) error. All results were assessed by one-way analysis of variance (ANOVA) and Duncan’s new multiple range test. A mixed model used to include the animal as a random factor in the analysis. A value of *p* < 0.05 was considered statistically significant.

## 3. Results and Discussion

### 3.1. Changes in Eating Quality of CSM during Freezing and Thawing

**Figure 1:**
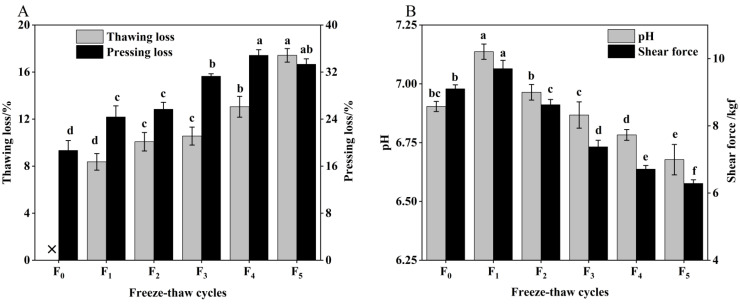
(**A**) Change in thawing loss, pressing loss and (**B**) pH, shear force of CSM during F-T cycles. F_0_, F_1_, F_2_, F_3_, F_4_, and F_5_: number of F-T cycles. ×: thawing loss did not occur when the number of F-T cycle was nil. Different letters (a–f) indicate significant differences (*p* < 0.05).

Meat quality refers to the overall standard of meat, including indicators such as pH, shear force, thawing loss, and pressing loss, which affect meat processing and consumption [18]. The changes in pH, shear force, thawing loss, and pressure loss of CSM during freeze–thaw (F-T) cycles are illustrated in Figure 1.

Thawing loss and pressing loss are important indicators to evaluate the water-holding capacity (WHC) of CSM [19]. As indicated in Figure 1A, the thawing loss and pressing loss increased significantly (*p* < 0.05) with the increase of F-T cycles. The values after one F-T cycle were 8.37% and 24.37%, respectively, and increased by 108.12% and 78.33% compared with the initial values, respectively. Water loss affects the appearance and weight of cattle rumen smooth muscle (CSM) [20]. Thawing loss and pressing loss of CSM result in reduced acceptability, due to the loss of amino acids or nucleotides [21]. This phenomenon can also be a result of protein oxidation (determined by sulfhydryl and carbonyl content) and lower pH during F-T cycles. Therefore, the regeneration and melting of ice crystals causes mechanical damage to cell membranes and muscle tissue, and the increased water loss leads to a decrease in the WHC of the muscle after the F-T cycle [22]. 

As illustrated in Figure 1B, pH first increased after a single F-T cycle and then fell. The pH was reduced significantly (*p* < 0.05) by 3.06% after five F-T cycles, which is primarily attributable to the lactic acid produced by anaerobic glycolysis and inorganic phosphate produced by ATP degradation, resulting in a drop in pH during the F-T cycle [2]. Therefore, multiple F-T cycles contributed to the reduction in pH of CSM. The shear force of CSM was significantly decreased by increasing F-T cycles (*p* < 0.05), which indicated that F-T cycles had a significant effect on muscle tenderness. Zhang et al. found that the shear force of meat is closely related to its muscle structure [23]. The muscle tissue damage induced by ice crystals during freezing was the major reason for the decline in CSM shear during the freeze–thaw cycle, a phenomenon consistent with the study by Qi et al. [24].

### 3.2. Changes in Scanning Electron Microscopy (SEM) of CSM during F-T Cycles

**Figure 2:**
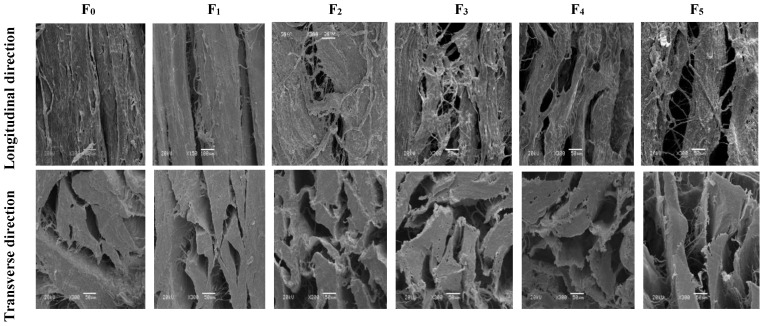
Changes in microstructure of transverse and longitudinal sections of CSM subjected to different F-T cycles. F_0_, F_1_, F_2_, F_3_, F_4_, and F_5_: number of F-T cycles.

The SEM of CSM is shown in Figure 2. In the longitudinal direction, contraction of the muscle fibers was evident, as evidenced by the formation of gaps. In addition, the degree of breakage of the muscle fibers increased with the increase of F-T cycles. Tan et al. reported that repeated F-T cycles caused drip loss and shrinkage of muscle fibers [25]. This might be related to protein degradation and the disruption by ice crystals [26]. In the transverse direction, muscle bundles were separated after five F-T cycles. The spaces between collagen fiber bundles became larger, more numerous, and disorganized, and the connective tissue was severely disrupted. F-T-induced protein denaturation can lead to a reduction in the structural denseness of muscle tissue, while ice formation during freezing strongly influences the aggregation and conformation of muscle protein structures which subsequently affect functionality [2]. The observed disruption of muscle fibers and the looser structure were consistent with lower shear force. The disruption of muscle structure by ice crystals, coupled with F-T-induced protein denaturation, is likely to be associated with reduced water-holding capacities of muscle, associated with higher thawing loss and pressing loss (Figure 1). Multiple F-T cycles accelerated the destruction of the CSM tissue microstructure.

### 3.3. Changes in Collagen Content and Solubility of CSM during F-T Cycles

**Figure 3:**
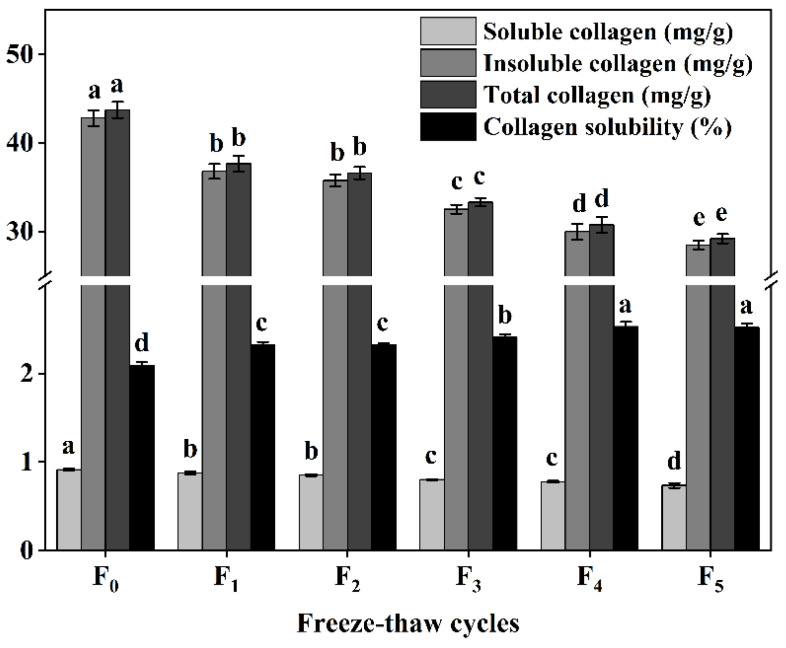
Changes in soluble collagen content, insoluble collagen content, total collagen content, and collagen solubility (%) of cattle CSM subjected to different F-T cycles. F_0_, F_1_, F_2_, F_3_, F_4_, and F_5_: number of F-T cycles. Different letters (a–e) indicate significant differences (*p* < 0.05).

Collagen content and solubility represent the main properties of collagen and are among the main factors affecting meat tenderness [11]. Our study also found that the content of collagen (soluble collagen, insoluble collagen, and total collagen) decreased significantly (*p* < 0.05) with the increase of F-T cycles (Figure 3), while the solubility of collagen increased significantly (*p* < 0.05) during F-T cycles (Figure 3). This can be attributed to the disruption of ice crystals, leading to degradation of proteoglycan CSM muscle-bundle membranes and muscle intima, and disruption of connective tissue membranes, increased solubility of collagen, and improved CSM tenderness, consistent with the study by Mikolajczak et al. [11].

### 3.4. Changes in Collagen Degradation of CSM during F-T Cycles

**Figure 4:**
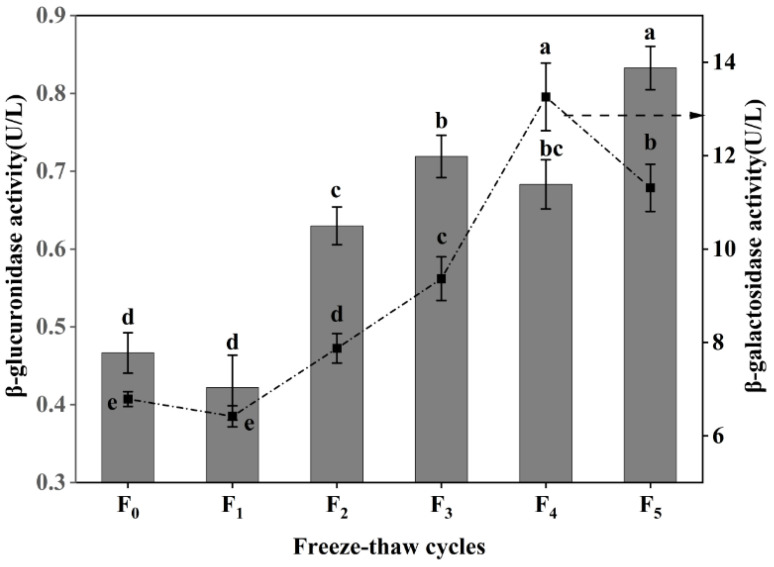
Changes in β-galactosidase activity and β-glucuronidase activity of CSM subjected to different F-T cycles. F_0_, F_1_, F_2_, F_3_, F_4_, and F_5_: number of F-T cycles. Different letters (a–e) indicate significant differences (*p* < 0.05).

β-galactosidase and β-glucuronidase are the key enzymes that degrade intramuscular collagen matrix polysaccharides [27], and the degradation of proteoglycan can improve the tenderness of muscle [28]. As shown in Figure 4, the activity of β-galactosidase increased at first and then decreased during F-T cycles, and reached the maximum (13.26 U/L) after four F-T cycles, a value which was significantly higher than that of frozen meat (6.79 U/L) (*p* < 0.05). However, the activity of β-galactosidase decreased to 11.31 U/L after five rounds of freezing and thawing. The β-glucuronidase activity showed a fluctuating increase (*p* < 0.05), with a 76.60% increase compared with frozen CSM at five F-T cycles. The increases in β-galactosidase activity and β-glucuronidase activity were due to the improvement of CSM tenderness after cattle were slaughtered, the heat emitted by various reactions, and the change of internal environmental pH, promoting the increase of β-galactosidase activity within a certain range [29]. The decrease in β-glucuronidase activity is attributed to the destructive effect of freezing and thawing on the enzyme itself [30]. The results showed that repeated F-T cycles promoted an increase in muscle β-galactosidase activity and β-glucuronidase activity, thereby accelerating the extent of CSM collagen degradation.

**Figure 5:**
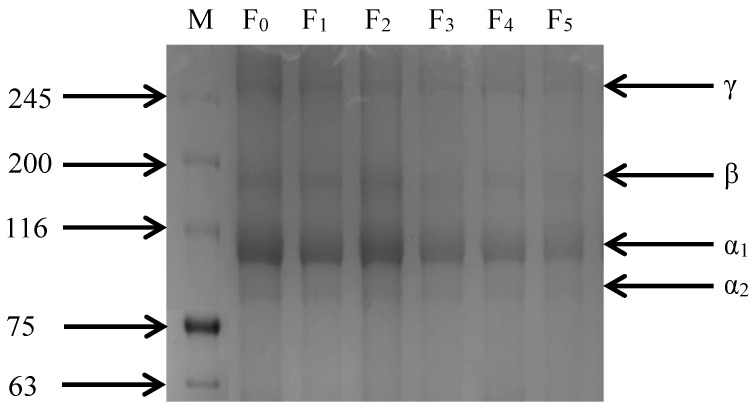
Changes in SDS-PAGE gel imaging of CSM collagen subjected to different F-T cycles. M: molecular weight of standard collagen (the numbers are in kDa); F_0_, F_1_, F_2_, F_3_, F_4_, and F_5_: number of F-T cycles.

As shown in Figure 5, the SDS-PAGE image of CSM collagen is similar to cattle Achilles tendon type I collagen, but with slightly different molecular weights [31]. CSM collagen exists in two different α-chains: α_1_ and α_2_. The gray value analysis of protein bands showed that the gray value of the α_1_ band was higher than that of the α_2_ band, indicating that the CSM collagen corresponded with the molecular composition of type I collagen. Furthermore, dimeric β-chains and trimeric γ-chains of the α-chain were observed. The four bands of collagen gradually darkened and there were no other small molecule protein bands during the F-T cycles, indicating that the purity of CSM collagen was high. Thus, repeated F-T cycles promoted the degradation of collagen, consistent with the results of β-galactosidase activity and β- glucuronidase activity. 

### 3.5. Changes in Collagen Secondary Structure of CSM during F-T Cycles

**Figure 6:**
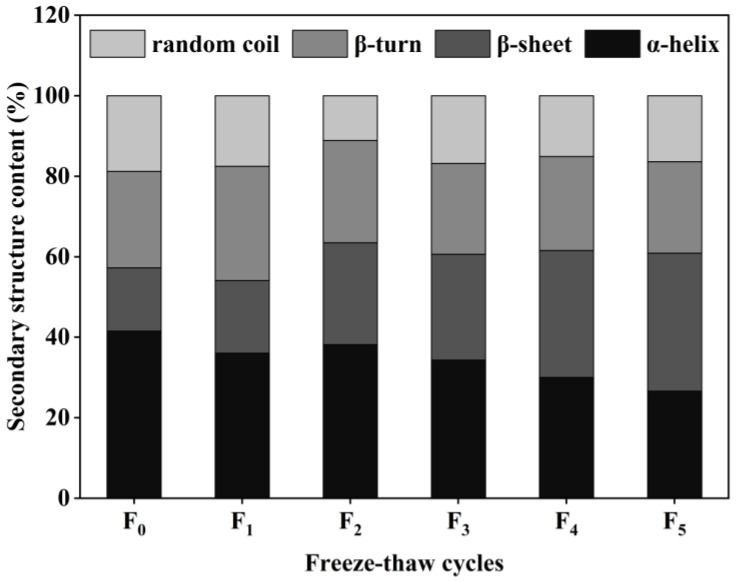
Changes in secondary structure content of CSM collagen subjected to different F-T cycles. F_0_, F_1_, F_2_, F_3_, F_4_, and F_5_: number of F-T cycles.

The secondary structure of proteins consists of four main forms: α-helix, β-sheet, β-turn, and random coil [32]. The content of collagen secondary structure in CSM samples was obtained by calculations using the software PeakFit 4.12. The results are shown in Figure 6. According to the form of the collagen secondary structure, the relative content of the α-helix in the control CSM collagen was 41.57%, and the relative content of the α-helix decreased to 26.64% after five F-T cycles. The relative amount of β-sheet in CSM collagen was significantly higher than in the control group (*p* < 0.05) during the F-T cycles. Changes in α-helix and β-sheet content depend mainly on the weakening of electrostatic interactions between amino acids, and the disruption of hydrogen bonds between the carbonyl oxygen and amino hydrogen in the polypeptide chain [33]. The results showed that the F-T treatment broke the hydrogen bonds that maintained the stable secondary structure of the protein, causing the structure of the protein to be stretched and to become disordered. Du et al. evaluated the effect of ice structuring protein induced by F-T processes on the microstructure and myofibrillar protein structure of mirror carp [34]. The results showed that the reduction in the α-helix content confirmed the decrease in the protein’s secondary structural order.

### 3.6. Changes in Collagen Thermal Stability of CSM during F-T Cycles

**Figure 7:**
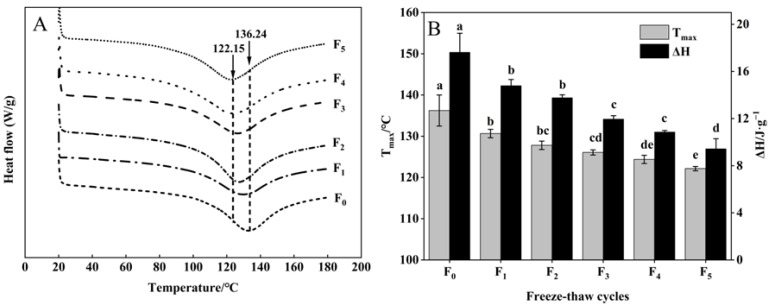
Changes in (**A**) DSC and (**B**) T_max_, ΔH of CSM collagen subjected to different F-T cycles. Tmax: denaturation temperature. ΔH: enthalpy. F_0_, F_1_, F_2_, F_3_, F_4_, and F_5_: number of F-T cycles. Different letters (a–e) indicate significant differences (*p* < 0.05).

The thermal stability of collagen of CSM is indicated by the thermal denaturation temperature (T_max_) and enthalpy (ΔH) of DSC [35]. As shown in Figure 7A, B, T_max_ emerged in the range of 122.15–136.24 °C. All samples showed significant decreases in T_max_ after five F-T cycles (*p* < 0.05). This phenomenon may be attributed to the disruption of the integral structure of collagen, forming a small subunit during F-T cycles [36], indicating a decrease in the thermal stability of CSM collagen.

The ΔH of samples declined during F-T cycles (Figure 7B). The decrease in collagen ΔH can be explained by collagen denaturation leading to disruption of the collagen structure and weakening of intramolecular hydrogen bonds during F-T cycles. The increase in ice crystal size and the emergence of recrystallization can severely damage the cell structure, which then leads to collagen denaturation during multiple F-T cycles [37]. Pan et al. [38] reported that F-T cycles induced changes in the thermal stability and structure of the protein. The results showed that F-T cycles reduced the thermal stability of collagen.

### 3.7. Changes in Collagen Emulsion Property, Surface Hydrophobicity, and Turbidity of CSM during F-T Cycles

**Figure 8:**
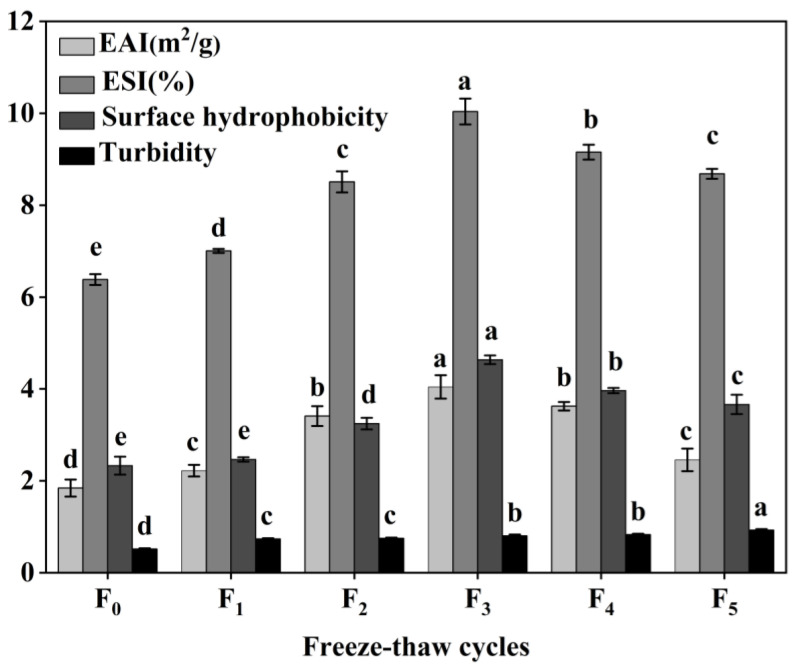
Changes in emulsifying properties, surface hydrophobicity and turbidity of CSM collagen subjected to different F-T cycles. F_0_, F_1_, F_2_, F_3_, F_4_, and F_5_: number of F-T cycles. Different letters (a–e) indicate significant differences (*p* < 0.05).

The functional properties of CSM collagen are shown in Figure 8. The emulsification properties of collagen are often evaluated by its EAI and ESI [39]. The initial EAI of collagen was 1.84 m^2^/g and reached a maximum value of 4.04 m^2^/g at three F-T cycles. The EAI dropped to 2.46 m^2^/g at five F-T cycle, which was still greater than the initial value (*p* < 0.05). The ESI values showed a tendency to rise and then over the F-T cycles. The results showed that F-T cycles significantly improved the EAI and ESI of CSM collagen samples, but the extent of this was dependent on the number of F-T cycles. It has been found that F-T cycles lead to a more disordered structure of CSM collagen, which improves its emulsification properties [40]. Cong et al. revealed a positive correlation between the emulsification properties of collagen and its surface hydrophobicity [39]. After F-T cycles, hydrophobic groups were exposed to denatured collagen, and collagen aggregation led to a decline in emulsification properties.

Surface hydrophobicity (H_0_) reflects changes in the surface microenvironment and internal structure of collagen [41]. In the present study, the H_0_ value of CSM collagen reached 4.64 at three F-T cycles, which was the expected result. Subsequently, the H_0_ value decreased to 3.67 after five F-T cycles were completed. However, the collagen H_0_ values after earlier F-T cycles were all better than the initial values (*p* < 0.05), despite the fact that the H_0_ values declined later during the F-T cycles. The increase in CSM collagen H_0_ values can be attributed to the exposure of hydrophobic side-chain groups and the rearrangement of molecules [42]. Thus, the H_0_ value of the CSM collagen rose and then fell over the complete F-T cycles. H_0_ is an important indicator of a protein’s ability to be absorbed through hydrophobic interactions on the oil–water interface, and is closely related to the emulsification properties of the protein [41]. Therefore, changes in H_0_ values can explain to some extent the changes in emulsification activity and emulsion stability.

The turbidity level of a protein solution is often utilized to characterize the degree of protein aggregation in the solution [17]. As shown in Figure 8, the turbidity of CSM collagen tended to increase during F-T (*p* < 0.05), indicating that the F-T treatment increased the degree of aggregation of bovine smooth muscle collagen. This may be because collagen is an amphiphilic molecule and the exposure of hydrophobic groups in its molecular structure causes rapid aggregation due to hydrophobic interactions [43].

## 4. Conclusions

In this study, we have illustrated the effects of different F-T cycles on collagen properties and quality of CSM. F-T cycles reduced the collagen content and water-holding capacity of CMS and improved its collagen emulsion properties, surface hydrophobicity, turbidity, and tenderness. The F-T cycle induced a decrease in thermal stability (Tmax, ∆H), pH, and water-holding capacity, and promoted the structural disorder of collagen. Therefore, those results show that CSM collagen structure changed and a decline in CSM quality occurred after F-T cycles, but its collagen functional properties were improved. Our study provides reference for the collagen properties of CSM during F-T cycles.

## Figures and Tables

**Table 1:** Live cattle and carcass traits.

Live or Carcass Traits	
Live weight	450 ± 50 kg
Average age	3 yr
Carcass weight	283.0 ± 64.0 kg
Subcutaneous fat thickness	14.0 ± 8.0 mm
Slaughter rate	54.0 ± 4.0%
Eye muscle area	62 ± 13 cm^2^

## Data Availability

Data is contained within the article.
